# The Effect of Calcium Gluconate on Platelet Rich Plasma Activation for VEGF-A Expression of Human Dental Pulp Stem Cells

**DOI:** 10.1055/s-0041-1735930

**Published:** 2021-12-22

**Authors:** Anggraini Margono, Dini Asrianti Bagio, Indah Julianto, Endang Suprastiwi

**Affiliations:** 1 Department of Conservative Dentistry, Faculty of Dentistry, Universitas Indonesia, Jakarta, Indonesia; 2 Department of Dermato Venereology, Faculty of Medicine, Universitas Sebelas Maret, Solo Surakarta, Indonesia

**Keywords:** platelet rich plasma, activation, calcium gluconate, calcium chloride

## Abstract

**Objective** Platelet-rich plasma (PRP) activation is an important factor in triggering the initial release of blood-derived growth factors from platelets. Vascular endothelial growth factor-A (VEGF-A) can be expressed by human dental pulp stem cells (hDPSCs) and plays an important role in dental pulp angiogenesis. The aim of this study is to analyze the effects of calcium gluconate on PRP activation in hDPSC VEGF-A expression.

**Materials and Methods** Two types of PRP and their corresponding activators were analyzed in this study: PRP (activated using calcium chloride/CaCl _2_ ) and PRP-T (activated using CaCl _2_ with the addition of 10% calcium gluconate). hDPSCs were obtained by using an out-growth method (DPSCs-OG), and harvest between the fifth and sixth passages, then cultured in three different media groups: control, PRP, and PRP-T, which were planted in 96 wells (5 × 10 ^3^ each well). The VEGF-A expression of hDPSCs was analyzed by using an ELISA test and observed at 24, 48, and 72 hours.

**Statistical Analysis** This study was performed by using one-way ANOVA ( *p* < 0.05) test.

**Results** There were significant differences between all groups ( *p* < 0.05) at 48 and 72 hours of observations, and no significant differences in the PRP and PRP-T groups at 48 and 72 hours of observations ( *p* > 0.05).

**Conclusion** PRP and PRP-T were equally effective in inducing VEGF-A expression of hDPSCs.

## Introduction

Platelet-rich plasma (PRP) has been widely studied as platelet-based secretomes in regenerative endodontics. 1 2 Studies have shown that PRP is effective for the proliferation, osteogenic differentiation and angiogenesis of human dental pulp stem cells (hDPSCs). 3 4 5 6 Several studies were attempted to modify PRP to human platelet lysate (hPL) by using the freeze-and-thaw method to promote growth factor (GF) release. 5 7 It was also shown that hPL from PRP has the potential to act as a medium for odontogenic differentiation of hDPSCs and stem cells of the apical papilla. 7 However, there are some limitations of the clinical application of PRP and its modification, especially due to the possibility of immune factor rejection, the complicated steps required in the process of modifying PRP into hPL, and its heterogeneous effect on cells (which reduces its ability to become a reservoir of blood-derived GF). 1 6 7 8

In addition, PRP requires a biological activator to release active GF from platelets. Activation with nonbiological chemicals can trigger the initial release of GF, but a proportion of the GF will remain in an inactive form, resulting in a disproportion between the high number of GF in PRP and the low number of active GF in cells. 5 8 The PRP activation method—using autologous thrombin, bovine, or calcium chloride (CaCl _2_ )—is performed to initiate blood clotting and fibrin scaffold formation. However, previous studies have shown that there may be unwanted side effects of using bovine (xenogenic) activators, side effects such as thrombosis, and a greater resulting concentration of antibodies. 8 It has also been reported that after activation, PRP significantly increases the concentration and number of GFs release as well as the rate of stabilization GF release. 8 9 10 11 12 Several studies have found that PRP plays a significant role in regulating inflammation and cell migration in regeneration processes after the activation process due to its ability to release blood-derived GF. 5 8 Platelets can carry about more than fifty α -granules that contain hundreds of bioactive proteins, including a wide range of pro-angiogenesis GF, such as vascular endothelial growth factor-A (VEGF-A) and fibroblast growth factor-2 (FGF-2), which play a role in regulating angiogenesis during healing processes and regeneration processes involving endothelial cells. 5 9

One previous study, performed using 3.2% calcium chloride (CaCl _2_ ) as a PRP activator (considered one of the safest activators), found that it does not cause side effects and is also highly cost-effective. 8 Other studies have also found that the use of autologous thrombin, obtained by the addition of 10% calcium gluconate to the PRP (known as platelet rich plasma-thrombin), may improve the quality of platelet gels and GF release. 13 The 10% concentration of PRP proven better than other concentration in their effect on most type of cells. 6 9 14

One of the most important blood-derived GFs in dental pulp regeneration is VEGF-A or VEGF. 15 16 17 VEGF has an important role in regulating the vascular permeability of pulp and the migration of pulp endothelial cells. During pulp inflammation, hDPSCs can release greater amounts of VEGF to regulate vascularization and angiogenesis. 18 19 This expression must therefore be regulated under the appropriate culture media in vitro, or it will diminish after 72 hours. 17 Vascular endothelial growth factor-A is a key factor in the dental pulp angiogenesis process and has a major impact on dental pulp regeneration. 15 16 17

The aim of this study was to evaluate the effect of PRP activators on the VEGF-A expression of hDPSCs. Two types of PRPs were used: PRP activated with CaCl _2_ and PRP-T activated with CaCl _2_ with an added of 10% calcium gluconate.

## Materials and Methods

### Human Dental Pulp Stem Cell culture

The method of isolating hDPCs used in this study was conducted by a previous study at the Prodia Stem Cells Laboratory. In the study, cells were recultured by using explant methods or out growth (DPSCs-OG) methods, 20 and harvested between the fifth and sixth passages. Twenty-four hours prior to the culture stage, cells in the three groups—the control group using Dulbecco's Minimal Essential Medium (DMEM), PRP group, and PRP-T group—were starved by reducing culture media from DMEM + FBS 20% to DMEM + FBS 1% (Cooper and Gonzalez-Hernandez, 2009). There is only one independent experiment with *n* = 3 for cell triplication (triplo), and three times of experimental observations (24, 48, and 72 hours). However, we use hDPSCs from nine patients which counted as biological replicates.

### Platelet Rich Plasma and Platelet Rich Plasma-Thrombin Preparation

**Fig. 1:**
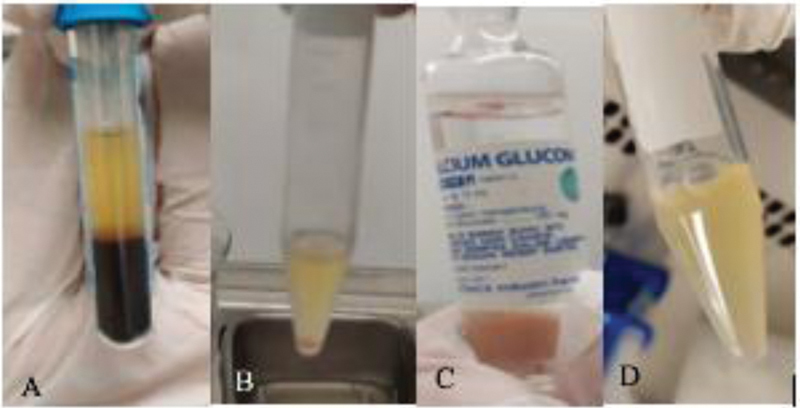
Vein cubitus blood after the first centrifugation. ( **A** ) shows the plasma layer, the buffy coat layer, and the PRP layer (which formed after the second centrifugation). ( **B** ) shows the addition of 10% calcium gluconate in the PRP-T tube. ( **C** ) shows the gel phase that forms 15 minutes after the procedure (at 37°C). PRP-T, platelet rich plasma thrombin.

PRP and PRP-T were taken from three healthy donors between 19 and 25 years old and was prepared according to the methods used by Franco et al. 13 About 10 mL of vein cubitus blood was collected and treated with a 3.2% CaCl _2_ anticoagulant. The tubes were first centrifuged at 400 g for 10 minutes. Three layers were then formed in the sample: the plasma layer, red blood cell layer, and the intermediate layer. Plasma and platelets form the top layer; red blood cells (RBC) form the bottom layer due to their density and heavy molecular weight; and the smooth and whitish intermediate layer is called the buffy coat layer and is made up of larger platelets and leucocytes ( Fig. 1A ). Once these layers had formed, the top layer was removed by using a Jelco 18 G needle. The buffy coat was extracted and divided into two separate tubes (this time without using an anticoagulant agent): one tube was used to produce plasma (PRP tube) and the other to produce thrombin (PRP-T tube). Only 1.5 mL of plasma was used to produce thrombin, added 0.5 mL of 10% calcium gluconate, and then waited for 15 minutes at 37°C for the gel phase to form. The two tubes were then centrifuged again at 800 g for 10 minutes. The two PRP and PRP-T tubes were then formed ( Fig. 1B–D ). The PRP (activated with CaCl _2_ ) and PRP-T (activated CaCl _2_ + 10% calcium gluconate) were diluted to a concentration of 10% by using DMEM. 13

### Viability Assay of Human Dental Pulp Stem Cells

The viability assay test for hPDSCs in this study used 96 wells (2 × 10 ^3^ cell/well) of 3-(4,5-dimethiazole-2-yl)2,5-diphenyltetrazolium bromide (MTT) assay read with a microplate reader at an absorbance of 595 nm on 24, 48, and 72 hours of observation, with one independent experiment.

### Vascular Endothelial Growth Factor-A Expression Analysis

The VEGF-A expression of hDPSCs was quantitatively analyzed by using a human VEGF-A ELISA kit (Elabscience, Wuhan Hubei) with a sensitivity of 18.75 pg/mL. The VEGF-A expression of hDPSCs was measured at 24, 48, and 72 hours on an ELISA microplate reader, under a wavelength of 450 nm, with one independent experiment.

### Statistical Analysis

Statistical analysis was conducted by using a one-way ANOVA and post hoc tamhane tests, with significance levels at 95% ( *p* < 0.05). Data were analyzed by using IBM SPSS Statistics Software, version 22.0 (IBM Corp., Armonk, New York, United States).

## Result

**Fig. 2:**
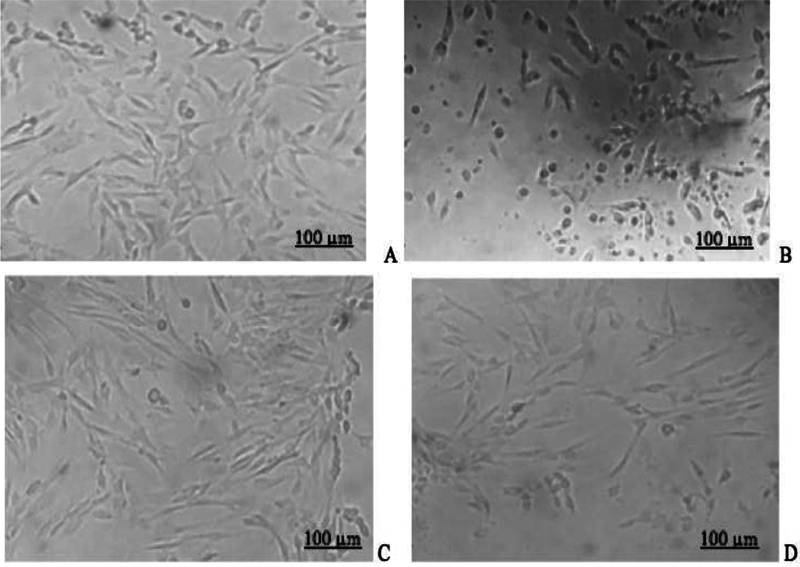
The hDPSCs morphology before ( **A** ) and after ( **B** ) starvation; hDPSCs in different cultured media; and PRP ( **C** ) and PRP-T groups ( **D** ) after 24 hours of cultured (scale of 100 μm) (Inverted microscope, Zeiss, Observer Z1, UK). hDPSC, human dental pulp stem cell; PRP-T, platelet rich plasma thrombin.

**Fig. 3:**
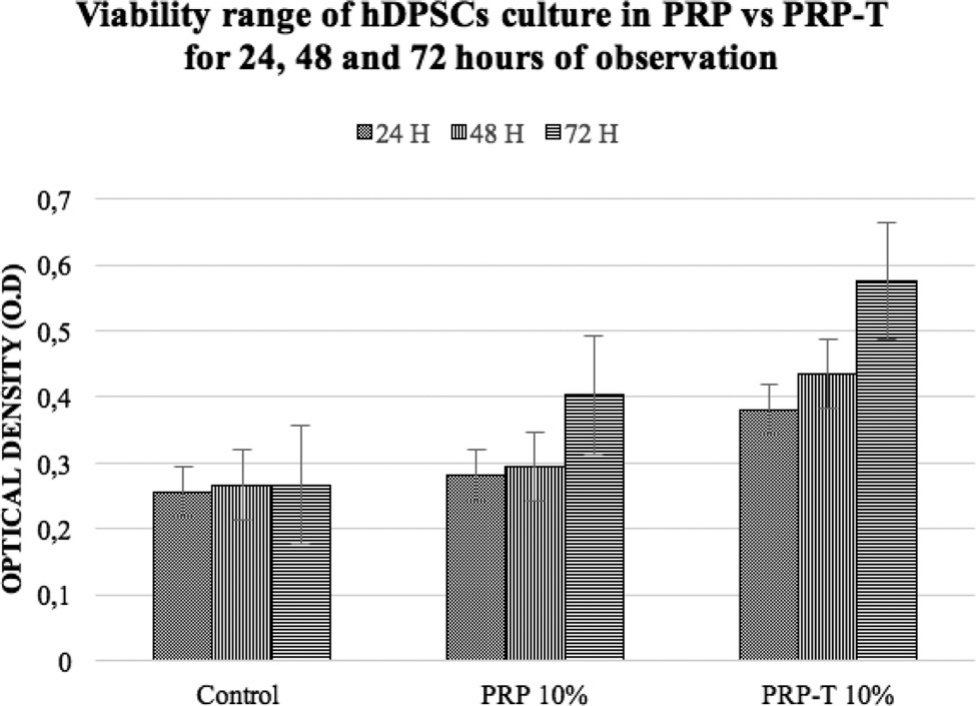
The viability range of human dental pulp stem cells cultured in PRP, PRP-T compare with control groups, showed an increase of optical density after 24, 48, and 72 hours of the PRP-T groups compare with PRP was statistically significant (one-way ANOVA test-post hoc Bonferroni;* *p* < 0.05). PRP-T, platelet rich plasma thrombin.

**Fig. 4:**
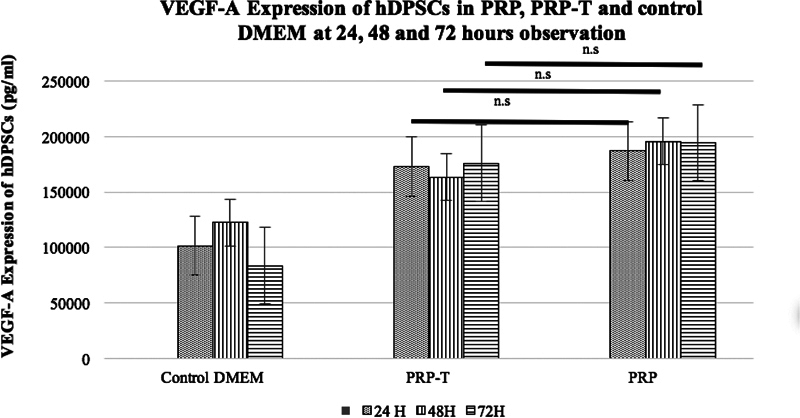
Quantitative result of post hoc vascular endothelial growth factor-A expression of hDPSCs in PRP and PRP-T groups compare with control group at 24, 48, and 72 hours of observation. Post hoc analysis showed statistically significant between PRP and PRP-T groups compare with control Dulbecco's minimal essential medium ( *p* < 0.05) at 24 and 72 hours of observation. There were no significant differences between PRP and PRP-T groups at 24, 48, and 72 of observation. (Post hoc LSD test; * *p* < 0.05). hDPSC, human dental pulp stem cell; n.s., not significant; PRP-T, platelet rich plasma thrombin.

The result of the flowcytometry analysis of hDPSCs showed phenotypes of stem cells with a positive cocktail of CD105 + , CD73+ and CD90+ for >70%, and a negative cocktail for <2% (Lin ^Neg^ ) (data not shown). Ultrastructural morphology of hDPSCs in three different culture media are shown in Fig. 2 . The viability of hDPSCs after culture in control and experimental groups (PRP and PRP-T) showed an increase of viable cells number related to time observations ( Fig. 3 ). The VEGF-A expression of hDPCs at 24, 48, and 72 hours is shown in Table 1 . There were significant differences between all groups (one-way ANOVA; *p* < 0.05) at 48 and 72 hours. The post hoc analysis VEGF-A expression of hDPSCs at 48 and 72 hours is shown in Fig. 4 .

**Table 1:** Vascular endothelial growth factor-A expression of human dental pulp stem cells in various culture media groups at the third time of observations

Culture media groups	Time of observation (ng/mL)		
	24 h	48 h	72 h
	Mean (SD)	Mean (SD)	Mean (SD)
Control	101,400 (25,709.92)	122,400 (15,524.17)	83,733.33 (25,146.23)
Blank VEGF	74,600 (6,082.76)	66,933.33 (577.35)	55,266.67 (3,511.88)
PRP group	173,066.67 (43,878.62)	163,333.33 (23.349,80)	176,066.67 (48,675.79)
PRP-T group	187,066.67 (41,860.88)	195,733.33 (36,692.41)	194,733.33 (56,011.90)
*p* -value	0.008 a	0.001 a	0.006 a

Abbreviations: PRP-T, platelet rich plasma thrombin; SD, standard deviation; VEGF, vascular endothelial growth factor.

a one way ANOVA test, *p* < 0.05.

After 24 hours of starvation, the hDPSCs displayed wrinkled ultrastructural morphology ( Fig. 2B ), distinct from their morphology prior to starvation ( Fig. 2A ). The hDPSCs morphology cultured in PRP and PRP-T groups showed similar characteristics: heterogeneous, spindle shape, and fibroblast shapes with minor elongation ( Fig. 2C, 2D ).

## Discussion

Platelets are a known reservoir of blood derived growth factor (GF) and cytokines can regulate the tissue healing process. 5 6 Platelet α -granules secrete bioactive molecules that are involved in cell proliferation, migration, and differentiation, and also contribute to pro- and anti-inflammatory processes. 21 A lack of standardized PRP preparation procedures and differences in PRP application may result in a heterogeneous effect on cells. 8 Of the steps needed to prepare PRP, platelet activation is one of the most crucial and may influence the availability of bioactive molecules and contribute to the generation of the tissue healing process. Platelet activation occurs as a result of a combination of fibrinogen cleavage, leading to fibrin mesh or platelet gel formation, and the externalization of α -granules, through the degranulation of platelets. These platelets were able to hold α -granules that contain hundreds of pro-angiogenic GF. 6 8 9 21

Calcium, as a second messenger in platelet activation, mediates platelet activation response. 8 Platelet activators are necessary inducers for producing platelet gel. 21 Changes in shape of plaletet, granule secretion, and aggregation are mediated by Calcium, but one research found that the release of direct Calcium on PRP may lead a coagulation. 22 23 Thrombin is the most effective platelet activator. 8 The presence of thrombin results in the generation of fibrin from fibrinogen and contributes to the formation and consolidation of the fibrin clot. In total, 3% of CaCl _2_ is the most frequently used activator and is observed to be the safest to use, as compared with bovine thrombin in the preparation of conventional PRP. 8 22 Studies have found that the generation of autologous thrombin can be induced by the addition of 10% calcium gluconate after the first spin of PRP preparation. This develops platelet rich plasma-thrombin (PRP-T) that has more dense gel phase form. 13 The addition of calcium gluconate can also induce greater release of GFs, which can be a good alternative for producing PRP and PRP-T using nonautologous tissue or animal products. 13 23 Blood-derived GFs can bind to the receptors of target cells after they have been released and then activate intracellular signals and pathways that induce essential components of wound healing, including cellular proliferation and matrix formation. 22 However, these GFs can also stimulate mesenchymal stem cell growth and endothelial cell migration, proliferation, and differentiation to improve angiogenesis and to modulate inflammation by promoting the chemotaxis of macrophages, monocytes, and polymorphonuclear cells 24 25

Previous studies have confirmed that the currently available commercial cell culture media, known as DMEM, permits the continuous growth of specific cell types without considering the effects of the metabolic environment of the original tissue. This non-serum condition resulted in a lack of metabolites, normally present in human fluids, that result in abnormal cell morphology and cell growth. 26 27 Therefore, the MTT Assay result showed an increasing viable cells number of hDPSCs in both PRP and PRP-T groups compare with control (DMEM). PRP-T groups showed higher viability rates of hDPSCs after 48 to 72 hours compare with PRP group.

The expression of hDPSCs VEGF-A was significantly different in cells that were cultured in the PRP and PRP-T groups compared with the control group at 48 and 72 hours ( Table 1 ). However, PRP group showed higher increase of hDPSCs VEGF-A expression compared with PRP-T group ( Table 1 ). This result was not in line with those previous study that demonstrated the VEGF-A expression of 10% PRP was decreased after 24 to 48 hours. Although, it was proved that 10 and 20% of hPL and 10% of PRP significantly increased cell viability of hDPSCs, especially at 48 hours. 5 This similar with the MTT assay result in this study ( Fig. 3 ). Another study showed that PRP released significantly higher concentrations of GF, but this release was not stable over longer period of time. 10 PRP therefore release a less total amount of GF compared with A-PRF at same period of time. Thus, A-PRF showed more stable and gradual release of GF compare with PRP. 11 This showed that PRP and PRP-T may induce VEGF-A expression of hDPSCs due to their ability to release high amounts of GF in various periods of time (12–72 hours). This was due to the heterogeneity in PRP protocol that might lead to a variety of PRP products that will create different biological and biochemical characteristics. 6 9 Although, most of the previous studies proved that PRP can act as a good reservoir of GFs for hDPSCs, but the uptake mechanism of GF release by PRP still remain unclear until now.

The present study showed that hDPSCs culture that used nonserum media expressed the lowest VEGF-A concentrations compared with other groups ( Fig. 4 ). 5 Other studies have shown that at 24 hours, CaCl _2_ and CaCl _2_ /thrombin produced significantly higher expressions of VEGF compared with collagen type I group ( *p* < 0.05). 8 This result is similar to the post hoc analysis of this study, which showed both PRP (activated with CaCl _2_ ) and PRP-T (activated CaCl _2_ + 10% calcium gluconate) hDPSCs express higher VEGF-A compared with the control group. Additionally, the PRP group showed a higher expression of hDPSCs VEGF-A compared with the PRP-T group. There were no significant differences in the hDPSCs VEGF-A expression of hDPSCs in PRP compared with PRP-T ( *p* > 0.05) at 48 and 72 hours of observation ( Fig. 3 ). This result was similar to the previous studies showed that there were no significant differences in GF expression resulting from the use of CaCl _2_ compared with thrombin as activators. 8

VEGF-A expression of hDPSCs at 24, 48, and 72 hours increased at equal levels when using CaCl _2_ , compared with using CaCl _2_ with an addition of 10% calcium gluconate to activate PRP. Nevertheless, the two activators induce the formation of the PRP gel phase at different times. The PRP (activated with CaCl _2_ ) took longer time to induce fibrin clot formation (up to 30 minutes) compare with PRP-T (activated CaCl _2_ + 10% calcium gluconate), which can result clot formation in 15 minutes ( Fig. 1 ). 8 13 The fibrin clot that formed through the use of CaCl _2_ was less dense than that formed by the addition of 10% calcium gluconate. 13 The 10% calcium gluconate in PRP-T can induce the release number of autologous thrombin and creates platelet rich fibrin (PRF). The calcium chloride inhibits citrate which allows the plasma to coagulate, while thrombin causes fibrin to polymerize and resulting a coagulated gel. 12 This fibrin products can induce neutrophil, increase cells migration, and it was a natural autologous scaffold that facilitate cells/tissue to regenerate. 28

These findings suggest that the selection of a PRP activator should be determined based on clinical need, whether as a cell growth scaffold or simply as a growth medium conditioned in regenerative endodontic therapy.

## Conclusion

Both PRP (activated with CaCl _2_ ) and PRP-T (activated with CaCl _2_ and the addition of 10% calcium gluconate) can induce VEGF-A expression of hDPSCs. Since they are equally effective in inducing VEGF-A expression of hDPSCs, further research needs to be conducted to evaluate other types of PRP-induced GFs that play an important role in angiogenesis and the regeneration of dental pulp.

**Ethical Approval**

This study was approved by the Ethical Committee of the Faculty of Dentistry, University of Indonesia (No.82/ethical approval/FKGUI/IX/2019; protocol number: 070940819). Informed consent was obtained from all participating adult subjects prior to the study.
